# A Two-Step Strategy to Enhance Activity of Low Potency Peptides

**DOI:** 10.1371/journal.pone.0110502

**Published:** 2014-11-12

**Authors:** Jamie R. Doyle, Benjamin N. Harwood, Subrahmanian Tarakkad Krishnaji, Vijay M. Krishnamurthy, Wei-En Lin, Jean-Philippe Fortin, Krishna Kumar, Alan S. Kopin

**Affiliations:** 1 Tufts Medical Center, Molecular Cardiology Research Institute, Molecular Pharmacology Research Center, Boston, Massachusetts, United States of America; 2 Program in Genetics, Sackler School of Graduate Biomedical Sciences, Tufts University, Boston, Massachusetts, United States of America; 3 Tufts University, Department of Chemistry, Medford, Massachusetts, United States of America; Medical School of Hannover, Germany

## Abstract

Novel strategies are needed to expedite the generation and optimization of peptide probes targeting G protein-coupled receptors (GPCRs). We have previously shown that membrane tethered ligands (MTLs), recombinant proteins comprised of a membrane anchor, an extracellular linker, and a peptide ligand can be used to identify targeted receptor modulators. Although MTLs provide a useful tool to identify and/or modify functionally active peptides, a major limitation of this strategy is the reliance on recombinant protein expression. We now report the generation and pharmacological characterization of prototype peptide-linker-lipid conjugates, synthetic membrane anchored ligands (SMALs), which are designed as mimics of corresponding MTLs. In this study, we systematically compare the activity of selected peptides as MTLs versus SMALs. As prototypes, we focused on the precursor proteins of mature Substance P (SubP) and Cholecystokinin 4 (CCK4), specifically non-amidated SubP (SubP-COOH) and glycine extended CCK4 (CCK4-Gly-COOH). As low affinity soluble peptides these ligands each presented a challenging test case for assessment of MTL/SMAL technology. For each ligand, MTLs and corresponding SMALs showed agonist activity and comparable subtype selectivity. In addition, our results illustrate that membrane anchoring increases ligand potency. Furthermore, both MTL and SMAL induced signaling can be blocked by specific non-peptide antagonists suggesting that the anchored constructs may be orthosteric agonists. In conclusion, MTLs offer a streamlined approach for identifying low activity peptides which can be readily converted to higher potency SMALs. The ability to recapitulate MTL activity with SMALs extends the utility of anchored peptides as probes of GPCR function.

## Introduction

The development of peptide ligands has increased over the past two decades in parallel with an expansion in the diversity of corresponding therapeutic targets, e.g. ion channels, pumps/transporters, enzymes, G protein-coupled receptors (GPCRs) [1]. Although approximately 40% of drugs in the clinical pipeline interact with GPCRs [1], only a small fraction of these receptors have been exploited as therapeutic targets [2]–[4]. Novel strategies to activate or block GPCRs are needed as tools to probe corresponding physiological functions and to validate additional receptors as potential drug targets.

**Figure 1:**
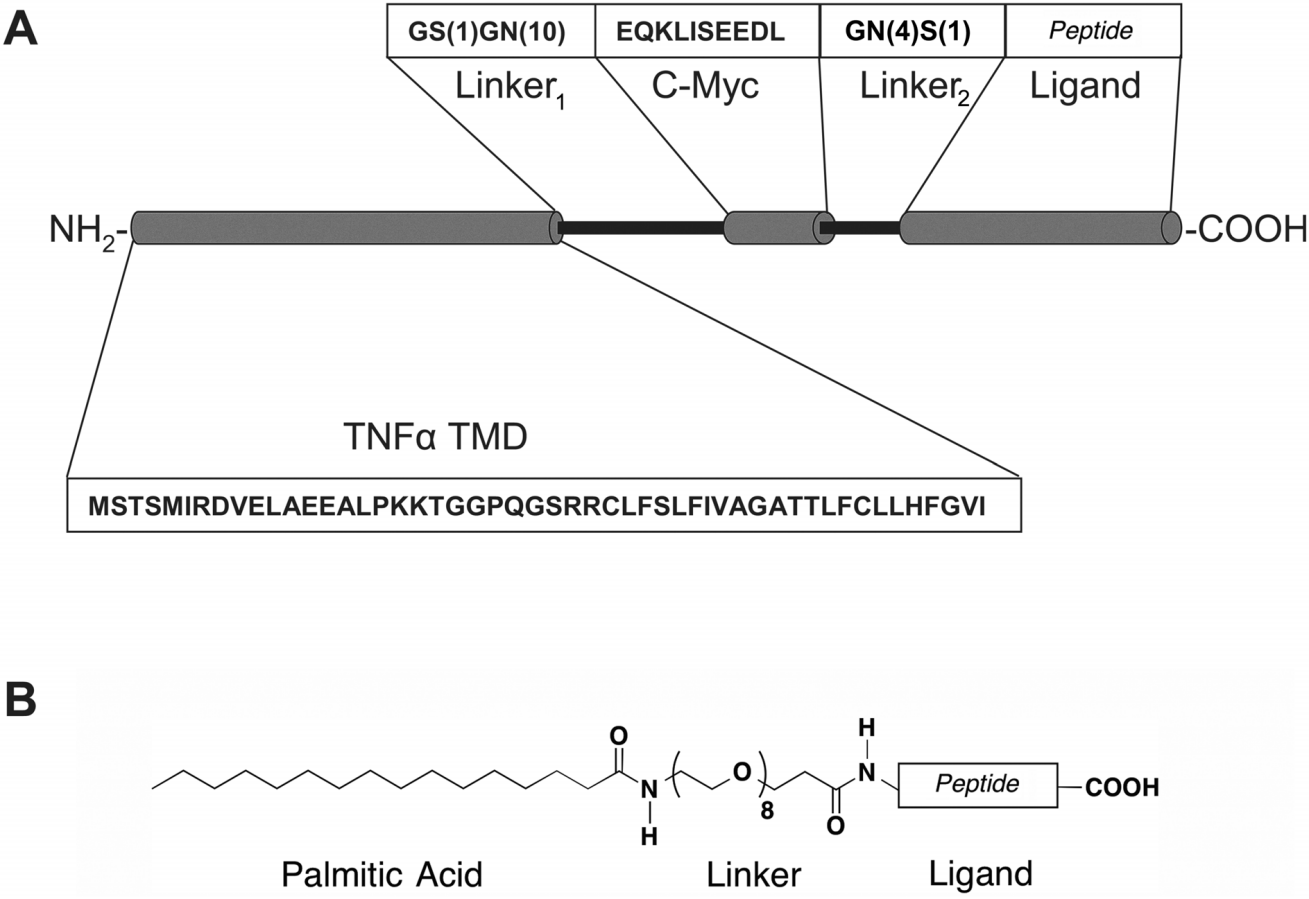
Cartoon models depicting a recombinant membrane tethered ligand (panel A) and a corresponding synthetic membrane anchored ligand (panel B). Note: Linker_2_ differs between tSubP and tCCK4 constructs. Tethered Substance P sequence is as shown, while the Linker_2_ sequence for tCCK4 constructs is GN(4)S(1)GS(9). Abbreviations: TNFα TMD = Tumor necrosis factor α transmembrane domain. Amino acids are represented in single letter code.

We have previously reported that membrane tethered ligands (MTLs) offer a novel approach to modulate GPCR activity both *in vitro* and *in vivo* [5]–[8]. An MTL complementary DNA (cDNA) encodes a single protein which includes a peptide ligand (localized outside the cell), an intervening linker, and a transmembrane domain (TMD) anchor (Figure 1A). Although the MTL approach allows ligands and subsequent modifications to be studied without the need for traditional peptide synthesis, a major limitation of this strategy is that it relies on delivery and expression of cDNA. The ability to optimize a construct using the MTL system and then utilize the corresponding peptide ligand as a synthetic membrane anchored ligand (SMAL, Figure 1B) would open a range of new possibilities for the use of such experimental probes.

To explore this possibility, we focused on two prototype peptide ligands, Substance P (SubP) and Cholecystokinin 4 (CCK4), both well characterized neuroendocrine hormones that activate selected cognate receptor subtypes. SubP is an eleven amino acid peptide that activates 3 neurokinin receptor subtypes: NK1, NK2, and NK3 [9], [10]. CCK4 is a tetrapeptide fragment of cholecystokinin that preferentially activates the cholecystokinin receptor subtype 2 (CCK2R) versus receptor subtype 1 (CCK1R) [11], [12]. The processing of CCK and SubP are similar with each existing as a C terminal glycine extended precursor protein. Following cleavage of the glycine residue, peptidylglycine α-amidating monooxygenase catalyzes the addition of a C-terminal amide group, thought to be important as both an affinity and efficacy determinant [13]–[16]. During the course of our initial pilot studies with these two peptides, we observed that non-amidated SubP and glycine extended CCK4 both demonstrated significant agonist activity as MTLs. These constructs provided tools to systematically examine how the pharmacological features of low potency soluble peptides compared when incorporated into a recombinant MTL versus a corresponding SMAL. Pharmacological features that were explored included receptor mediated activity, subtype specificity, and the susceptibility to inhibition by known antagonists of corresponding free peptides.

Our results suggest that MTLs offer an expedited approach to screen for low activity peptides that will have enhanced function as SMALs. Once identified as an active MTL, SMALs offer the possibility of direct administration rather than recombinant expression. This two-step strategy may be utilized to enhance the identification and optimization of a novel class of GPCR probes, i.e. MTLs that can be easily administered as SMALs.

## Materials and Methods

### Cell Culture and Transfections

Human embryonic kidney cells (HEK293) were maintained at 37°C in a humidified 5% CO_2_ atmosphere and cultured with Dulbecco’s modified Eagle’s medium (Invitrogen, Chicago, IL) containing 10% fetal bovine serum, 100 U/mL penicillin, and 100 µg/mL streptomycin. Cells were seeded into 96-well plates and grown to ∼80% confluence. Cells were transfected for 24 hours using polyethylenimine (Sigma, Atlanta, GA) in serum-free medium [17] with cDNAs encoding, a) tethered ligand (where noted), b) 3 ng of indicated receptor, c) 25 ng of a 5X-SRE-Luc-pest (pGL4.33), a luciferase reporter construct under the control of a serum response element (Promega, Madison, WI), and d) 10 ng of β-galactosidase to control for transfection efficiency.

### Plasmids

Neurokinin receptors were purchased from the Missouri S+T cDNA Resource center (Rolla, MO). The CCK2 receptor was cloned as previously reported [18]; the CCK1R cDNA was PCR amplified based on a published sequence [19]. Each receptor cDNA encoding the human sequence was subcloned into pcDNA1.1. Tethered SubP and CCK4 constructs were generated using an MTL with a type II transmembrane domain as a template cDNA which results in a free extracellular C-terminus of the corresponding peptide (Figure 1) [8]. Oligonucleotide-directed, site-specific mutagenesis was used to introduce sequences encoding the following peptides with a free carboxy terminus: SubP, RPKPQQFFGLM and CCK4, WMDF. For the glycine extended construct, the corresponding oligonucleotide encoded an additional glycine residue at the C-terminus of the CCK4 peptide (i.e. WMDFG). The nucleotide sequences of all receptor and tethered ligand coding regions were confirmed by automated DNA sequencing and analyzed using Vector NTI software (Invitrogen, Chicago, IL). The expression of membrane tethered Substance P, CCK4, and CCK4-Gly was assessed by ELISA using an antibody directed to the myc epitope tag within the MTL cDNA construct [8]. Three independent experiments indicated that each MTL was expressed at the cell surface (data not shown).

### Peptides

**Figure 2:**
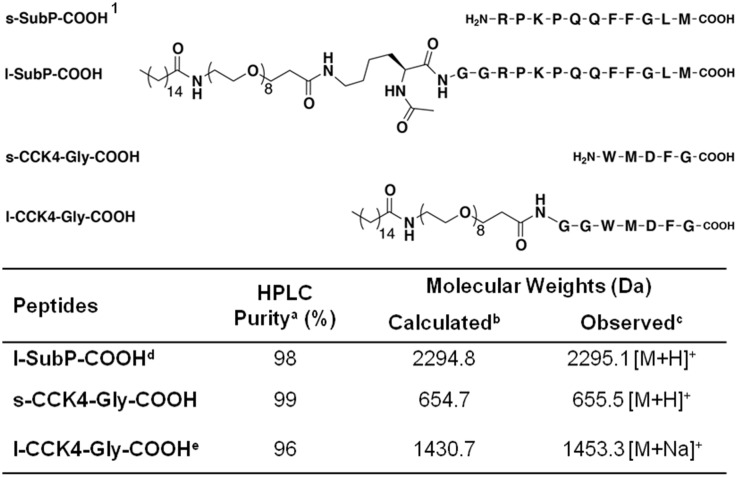
Chemical structure, purity, and molecular weight of synthesized peptides. a) Purity as determined by analytical RP-HPLC [Vydac C18, 5 ìm, 4 mm×250 mm] using a binary solvent system [A: H_2_O/CH_3_CN/TFA (99/1/0.1); B: CH_3_CN/H_2_O/TFA (90/10/0.07)] with a linear gradient of 65–80% solvent B over 20 min. The flow rate was set at 1 mL/min and elution was monitored by absorbance at 230 nm. b) Expected molecular weights were calculated using Peptide Mass Calculator v3.2. c) Observed molecular weights as determined using MALDI-TOF MS in reflectron positive mode using α-cyano-4-hydroxycinnamic acid as the matrix. d) Acetylated lysine GG spacer (Ac-Lys-GG). e) GG spacer coupled to the *N*-terminus of the peptide before pegylation. ^1^s-SubP-COOH peptide: purchased commercially from American Peptide Company.

The following peptides were purchased from American Peptide Company (Sunnyvale, CA): s-SubP-NH_2_, s-SubP-COOH, s-CCK4-NH_2_, and sulfated s-CCK8-NH_2_. All other peptides listed in Figure 2 and Figure S1 were synthesized using the *in-situ* neutralization protocol for *t*-Boc chemistry [20]. Synthesis was carried out on a 0.5 mmol scale on 4-hydroxymethyl-phenylacetamidomethyl (PAM) resin for l-SubP-COOH, s-CCK4-Gly-COOH and l-CCK4-Gly-COOH; and on *p*-methylbenzhydrylamine (MBHA) resin for l-SubP-NH_2_ and l-CCK4-NH_2_. Amino acids were used with the following side chain protecting groups: Arg(Tos), Asp(OBzl), Gln(Xan), Lys(Fmoc), Lys(2-Cl-Z) and Trp(For). Peptide coupling reactions were carried out with a 4-fold excess (2.0 mmol) of activated amino acid for at least 15 min. The *t*-Boc protecting group on the *N*-terminus was removed using trifluoroacetic acid (TFA). The PAM resin from the CCK4 peptide synthesis was split into two equal portions. One portion of the resin was used for synthesizing non-lipidated peptides. The CCK4 (s-CCK-Gly-COOH) peptide was left unmodified on the *N*-terminus. This peptide served as the positive control for the lipidated counterparts. The second portion of the CCK4 and SubP peptide on PAM, and the CCK4 and SubP peptide on the MBHA resins were modified on solid support as follows to yield test lipidated peptides (l-CCK4-Gly-COOH, l-SubP-COOH, l-CCK4-NH_2_ and l-SubP-NH_2_). Spacers (these are amino acids used between the polyethylene glycol,PEG, linker and the peptide of interest) were introduced on the peptides before PEGylation (Ac-Lys-GG for SubP and GG for CCK4). The *N*-terminus of the peptides on resin, and the *N*-*t*-Boc group on the GG spacer for CCK peptides were deprotected with TFA, and the *N* ^ε^-Fmoc side chain protection of the Lys-GG spacer for SubP peptides with 10% piperidine in DMF (N,N-Dimethylformamide). The deprotected *N*-terminus was PEGylated with *N*-Fmoc-PEG8-propionic acid using standard HBTU (*N*,*N*,*N*′,*N*′-Tetramethyl-O-(1 H-benzotriazol-1-yl)uroniumhexafluorophosphate) coupling conditions. The *N*-Fmoc protecting group on the PEG linker was removed by treatment with 10% piperidine in DMF for 5 min. Palmitic acid was subsequently conjugated to the *N*-terminal amine of the PEGylated peptide. The *N* ^α^-*t*-Boc protecting groups on the Lys-GG spacers of SubP peptides were deprotected and acetylated (7∶2∶1 of DMF:Ac_2_O:Pyridine) in the case of SubP peptide on PAM resin, or coupled with 4-Chloro-7-nitro-1,2,3-benzoxadiazole (NBD-chloride) in 9∶1 DMF:DIEA (N,N-Diisopropylethylamine) in the case of SubP peptide on MBHA. Peptides were cleaved from the resin using high HF conditions [21] with minor modifications applied to the literature procedure. For the SubP peptide, longer reaction times were employed to ensure complete removal of the Arg(Tos) protecting group (90% anhydrous HF/10% anisole at 0°C for 2 h). For the CCK4 peptides, 1,3-Propanedithiol (PDT) was used in the HF cleavage mixture to ensure deprotection of the formyl protecting group and prevent oxidation of methionine to its sulfoxide derivative: 85% anhydrous HF/10% anisole/5%PDT at 0°C for 2 h [22]. Following cleavage from the resin, and evaporation of HF, crude peptide products were precipitated and triturated with cold Et_2_O. Unmodified peptides were extracted using 10% AcOH in H_2_O and the lipidated peptides were extracted using 10% AcOH in H_2_O followed by 10% AcOH in 50% EtOH/H_2_O. Crude peptides were purified by RP-HPLC using the following solvent composition: solvent A, H_2_O/CH_3_CN/TFA (99/1/0.1); solvent B, CH_3_CN/H_2_O/TFA (90/10/0.07) [Vydac C18, 10 µm, 22 mm×250 mm]. The purities of the peptides were assessed by analytical RP-HPLC [Vydac C18, 5 µm, 4 mm×250 mm]. The molar masses of peptides were determined by MALDI-TOF MS. Peptide concentrations in the CCK series were determined using tryptophan absorbance (ε = 5580 M^–1^·cm^–1^ at 278 nm) [23] and l-SubP-NH_2_ concentration was determined using NBD absorbance (ε = 19000 M^–1^·cm^–1^ at 460 nm). The concentration of the l-SubP-COOH was measured using amino acid analysis (Molecular Biology Core Facility at Dana-Farber Cancer Institute, Boston, MA). The lipidated SubP peptides (l-SubP-COOH and l-SubP-NH_2_) included a KGG attachment coupled to the *N*-terminus to allow attachment of the corresponding PEG8 plus palmitic acid to the side chain *ε*-amine of lysine. Subsequent to conjugation of the linker and lipid, the *t*-Boc protecting group on the *α*-amino group of lysine was removed and acetylated to yield the *N*-α-acetyl-L-Lys-GG spacer. In comparison, the lipidated CCK4 analogues (l-CCK-Gly-COOH and l-CCK4-NH_2_) contained only a GG spacer used for subsequent attachment of the anchor. A general scheme of lipidated peptides is illustrated in Figure 1B. Detailed chemical structures, purities, and molecular weights of the synthetic peptides are shown in Figure 2 and Figure S1.

### Assessment of Ligand Activity

Tethered ligand induced signaling was assessed in HEK293 cells 24 hours after transfection. For soluble and lipidated peptides, 20 hours following transfection, cells were stimulated in serum-free medium for an additional 4 hours. The activity of all experimental ligands (MTLs and SMALs) was compared to a 4 hour treatment of receptors with amidated endogenous ligands: s-SubP-NH_2_ for NK1-3R, s-CCK4-NH_2_ for CCK2R, and sulfated s-CCK8-NH_2_ for CCK1R. For antagonist assays, CP 99994 or YM022 (Tocris, Minneapolis, MN) were added concurrently with soluble agonist for 4 hours. With tethered ligands, antagonists were added 4 hours after transfection; activity was assessed following an additional 20 hour incubation. Quantification of luciferase and β-galactosidase activities were performed as previously described [6]. Data were analyzed by nonlinear curve fitting using Graph Pad Prism 5.0 software.

## Results

Although amidated SubP and CCK4 are well characterized peptides, fewer studies have examined the activity of precursor forms of these hormones. These low potency ligands provide useful tools to investigate how membrane anchoring can influence activity. In pursuing this objective, we have focused this study on elucidating the pharmacological properties of non-amidated SubP and glycine extended CCK4 as freely soluble peptides versus tethered and lipidated counterparts.

**Figure 3:**
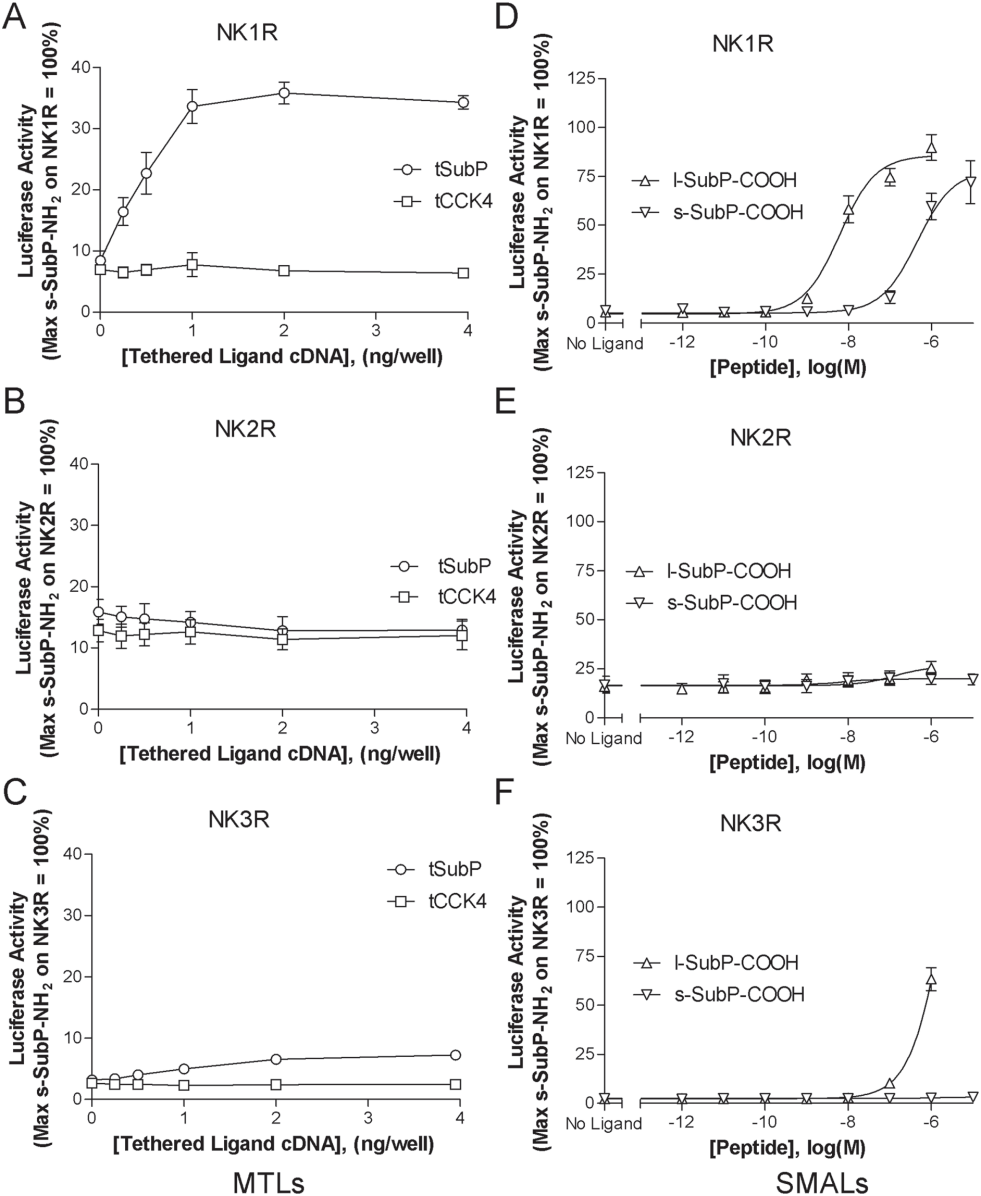
Recombinant SubP MTL mediated signaling predicts activity of a corresponding SMAL on neurokinin receptors. Both tSubP and l-SubP-COOH activate NK1R (panels A and D) and NK3R (panels C and F) with no observed activity at NK2R (panels B and E). HEK293 cells were transiently cotransfected for 24 hours with cDNAs encoding: the designated NK receptor subtype, a 5X-SRE-Luc-pest reporter construct (pGL4.33), tethered ligand (for MTL assays, left panels), and a β-galactosidase gene to control for transfection variability. For assessment of SMAL induced signaling, cells were stimulated with ligand for an additional 4 hours. Luciferase activity was quantified and normalized relative to a 4 hour stimulation with 1 µM soluble amidated substance P (s-SubP-NH_2_) on the corresponding NK receptor subtype. Data represent the mean ± SEM from 3 independent experiments, each performed in triplicate. Abbreviations: tSubP, tethered Substance P; tCCK4, tethered CCK4; s-SubP-COOH, soluble Substance P with a C-terminal free acid; l-SubP-COOH, lipidated Substance P with a C-terminal free acid; NK1R, neurokinin 1 receptor; NK2R, neurokinin 2 receptor; and NK3R, neurokinin 3 receptor.

**Figure 4:**
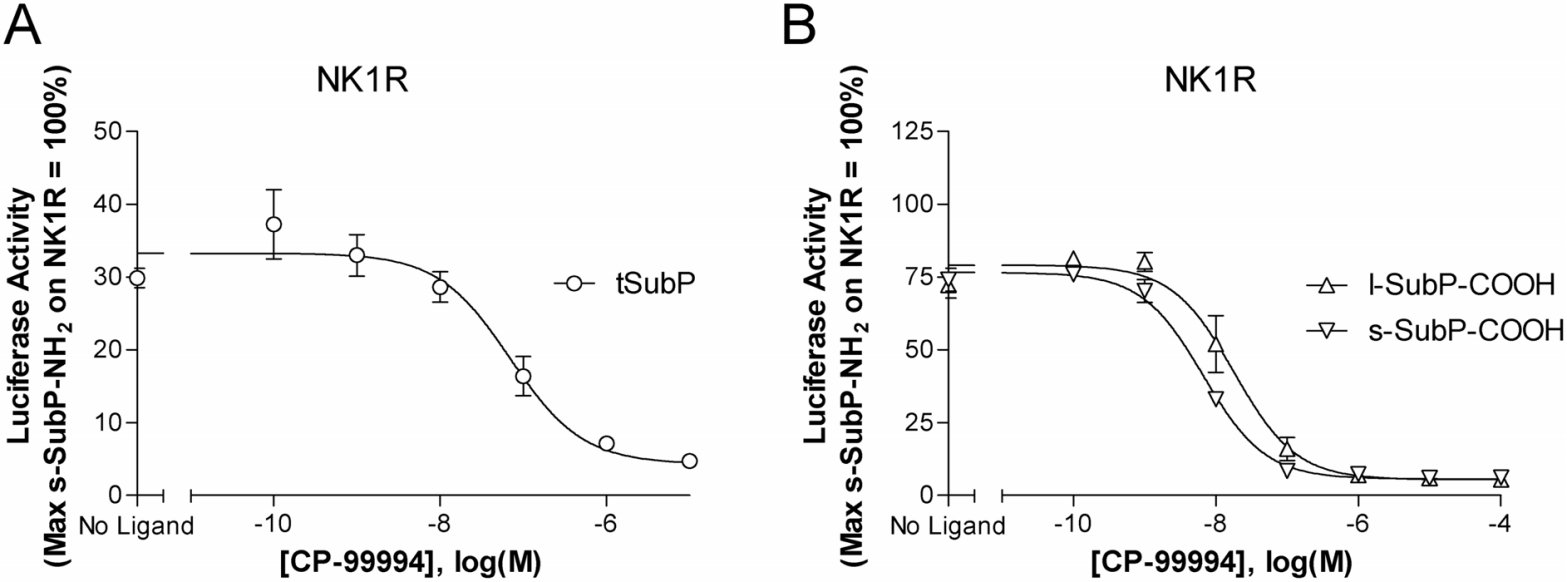
CP 99994 inhibits NK1R signaling induced by either a recombinant SubP MTL, soluble SubP with a C-terminal free acid (s-SubP-COOH), or the corresponding SMAL (l-SubP-COOH). A small molecule, CP 99994, inhibits NK1R activation by tSubP (panel A), s-SubP-COOH and l-SubP-COOH (panel B). HEK293 cells were transiently cotransfected with cDNAs as outlined in Materials and Methods. For tSubP experiments (panel A), 4 hours following transfection, cells were treated with increasing concentrations of CP 99994 for 20 hours. For s-SubP-COOH and l-SubP-COOH experiments (panel B), 20 hours after transfection cells were treated with increasing concentrations of CP 99994 and 1 µM of indicated soluble ligands for an additional 4 hours. Luciferase activity was quantified and normalized relative to a parallel preparation of NK1R expressing cells stimulated for 4 hours with s-SubP-NH_2_ (1 µM). Data represent the mean ± SEM from 3 independent experiments, each performed in triplicate.

We initiated our study with investigations focused on non-amidated SubP (SubP-COOH) as a recombinant MTL (tSubP). Activity of this construct was examined on each of the three human neurokinin receptor subtypes. When coexpressed with either NK1 or NK3 receptor, tSubP led to a cDNA concentration dependent increase in receptor mediated signaling (Figure 3A and C) whereas tSubP did not activate the NK2R (Figure 3B). In contrast, as a freely soluble ligand, s-SubP-COOH activated only the NK1R (Figure 3D, E, F). Efficacy/potency comparisons were then carried out using a corresponding SMAL, a SubP peptide with the addition of a PEG linker and a palmitic acid at the amino terminus, i.e. lipidated SubP-COOH (l-SubP-COOH). This synthetic lipidated peptide mimicked the pharmacological activity of its genetically engineered tethered counterpart (tSubP). Both NK1 and NK3 receptors were activated by l-SubP-COOH (Figures 3D and F). When assessed at the NK2R, no signaling was observed (Figure 3E). Comparison of soluble and lipidated-SubP-COOH at the NK1R (Figure 3D) revealed that the lipidated analog had enhanced potency; corresponding EC_50_ values reported in Table 1 are as follows: l-SubP-COOH (EC_50_ = 6.1 nM) and s-SubP-COOH (EC_50_ = 449.3 nM). To further probe the pharmacological properties of MTL and SMAL induced receptor activation, we assessed the ability of a non-peptide inhibitor to block NK1R mediated signaling. CP 99994, a small molecule neurokinin receptor antagonist [24], [25], inhibited signaling by soluble, MTL, and SMAL forms of SubP. As illustrated in Figure 4A, tSubP activity is inhibited with an IC_50_ of 69.5 nM. Agonist activities of l-SubP-COOH and s-SubP-COOH were also effectively blocked by CP 9994 (Figure 4B) with IC_50_ values of 18.0 nM and 6.7 nM, respectively.

**Table 1:** Half-maximal effective concentration (EC_50_) for selected ligands at NK1R or CCK2R.

	NK1R		CCK2R
Ligand	EC_50_ (nM)	Ligand	EC_50_ (nM)
s-SubP-NH_2_	1.4	s-CCK4-NH_2_	4.9
l-SubP-NH_2_	0.4	l-CCK4-NH_2_	2.6
s-SubP-COOH	449.3	s-CCK4-Gly-COOH	>1000
l-SubP-COOH	6.1	l-CCK4-Gly-COOH	>1000

All values represent the mean from at least three independent experiments, each performed in triplicate.

**Figure 5:**
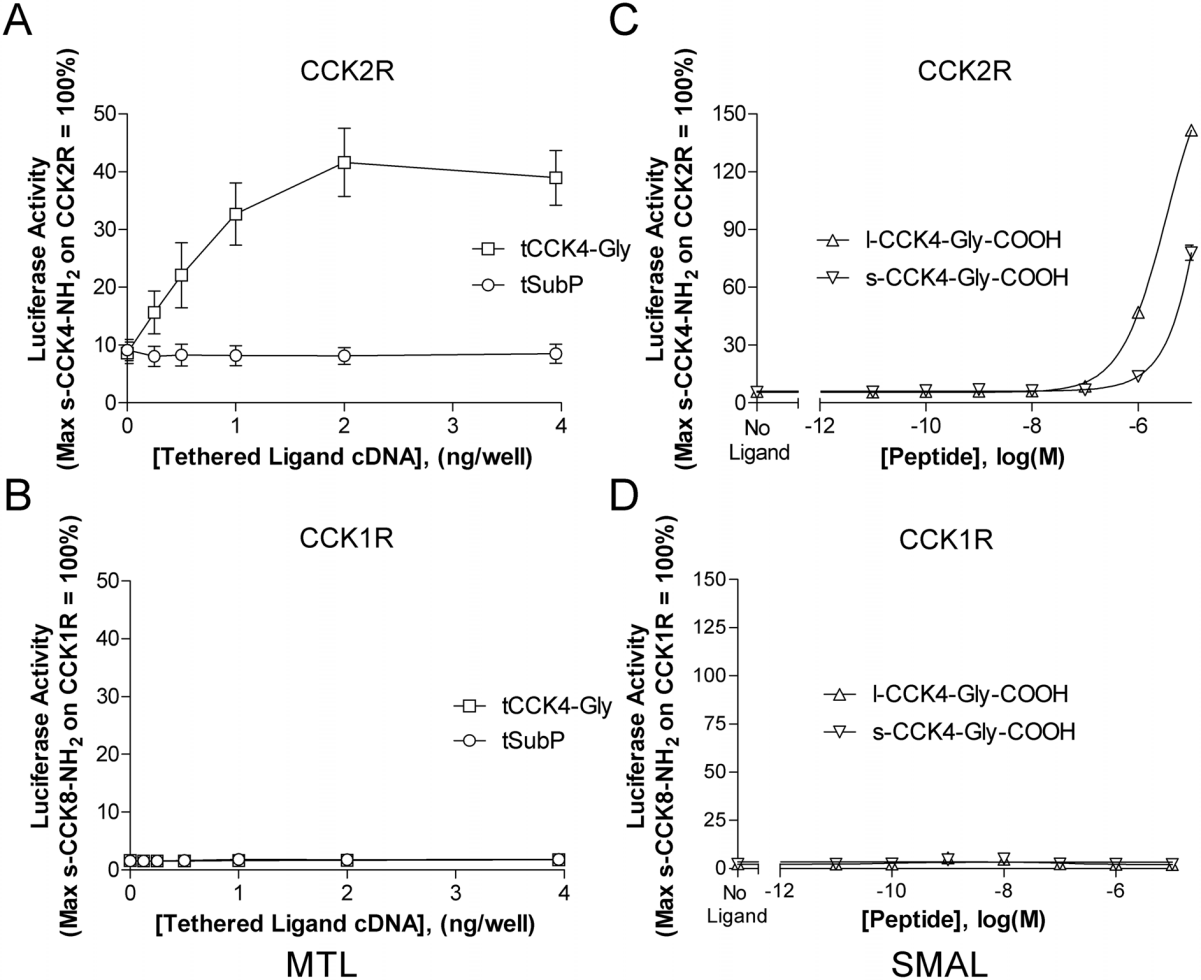
Tethered glycine extended CCK4 (tCCK4-Gly) anticipates activity of the corresponding synthetic membrane anchored ligand. Tethered CCK4-Gly activates the CCK2 receptor (panel A). Potency of the corresponding lipidated SMAL (l-CCK4-Gly) exceeds that of soluble CCK4-Gly (panel C). CCK4-Gly as a tethered, soluble or lipidated ligand fails to activate the CCK1 receptor (panels B and D). HEK293 cells were transiently cotransfected with cDNAs encoding: the designated CCK receptor subtype, a 5X-SRE-Luc-pest reporter construct (pGL4.33), tethered ligand (as indicated) and a β-galactosidase gene to control for transfection efficiency. Tethered ligand activity was measured 24 hours following transfection. To assess activity of soluble and lipidated CCK4-Gly, cells were stimulated for an additional 4 hours with ligand. Both soluble and tethered ligand activity was quantified relative to a parallel preparation of CCK receptor expressing cells stimulated for 4 hours with soluble amidated CCK4 (s-CCK4-NH_2_, 10 µM) for CCK2R or soluble sulfated/amidated CCK8 (s-CCK8-NH_2_, 10 µM) for CCK1R. Data represent the mean ± SEM from 3 independent experiments, each performed in triplicate. Abbreviations: tCCK4-Gly, tethered glycine extended CCK4; tSubP, tethered Substance P; s-CCK4-Gly-COOH, soluble glycine extended CCK4 with a C-terminal free acid; l-CCK4-Gly-COOH, lipidated glycine extended CCK4 with a C-terminal free acid; CCK2R, cholecystokinin 2 receptor; CCK1R, cholecystokinin 1 receptor.

**Figure 6:**
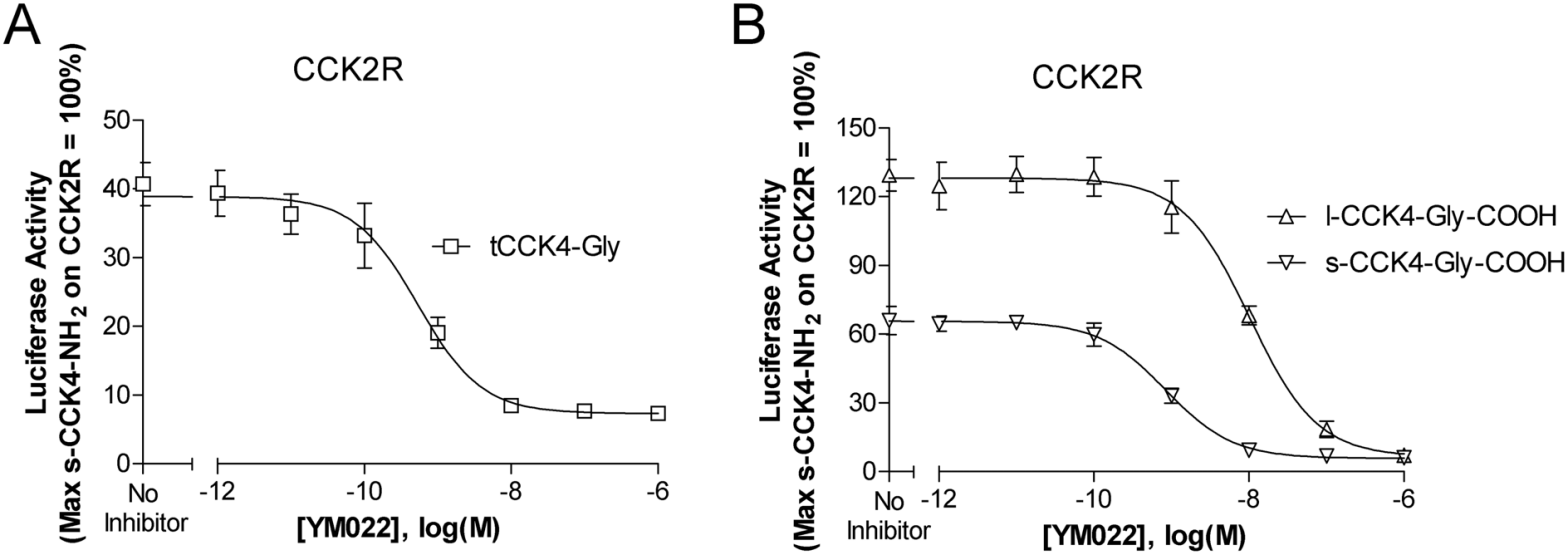
YM022 inhibits CCK2R signaling induced by tethered CCK4-Gly, soluble CCK4-Gly, or lipidated CCK4-Gly. A) YM022 blocks tethered CCK4-Gly mediated CCK2R signaling. HEK293 cells were cotransfected with cDNAs encoding: CCK2R, a 5X-SRE-Luc-pest reporter construct, tCCK4-Gly (as indicated), and a β-galactosidase gene to control for transfection efficiency. Four hours following transfection, cells were with treated with increasing concentrations of YM022 for 20 hours. Luciferase activity was quantified and normalized relative to a parallel preparation of CCK2R expressing cells stimulated for 4 hours with soluble amidated CCK4 (s-CCK4-NH_2_, 10 µM). B) YM022 blocks s-CCK4-Gly-COOH and l-CCK4-Gly-COOH mediated activation of CCK2R. HEK293 cells were transfected as indicated above. Twenty hours after transfection, cells were with treated with increasing concentrations of YM022 together with either 10 µM of l-CCK4-Gly-COOH or s-CCK4-Gly-COOH. Following an additional four hour stimulation, luciferase activity was quantified and normalized as outlined for panel A. Data represent the mean ± SEM from 3 independent experiments, each performed in triplicate. Abbreviations: tCCK4-Gly, tethered glycine extended CCK4; s-CCK4-Gly-COOH, soluble glycine extended CCK4 with a C-terminal free acid; l-CCK4-Gly-COOH, lipidated glycine extended CCK4 with a C-terminal free acid; CCK2R, cholecystokinin 2 receptor.

In addition to studying how membrane anchoring influences SubP activity, we also evaluated a second low potency peptide, glycine extended CCK4 (CCK4-Gly). Like SubP, the mature CCK4 peptide is also endogenously α amidated at the C-terminus. CCK4-NH_2_ is a well-established CCK2R ligand [12], [18]. In preliminary experiments, membrane tethered CCK4 (tCCK4), minimally activated CCK2R (data not shown). In contrast to tCCK4, we noted that with addition of a C-terminal glycine residue (tCCK4-Gly), activity of this construct at the CCK2R significantly increased. As illustrated in Figure 5A, tCCK4-Gly activated the CCK2R in a cDNA concentration dependent manner. In contrast, this construct showed no activity on the CCK1R (Figure 5B). To determine if the activity of the corresponding lipidated peptide would again (as with SubP) parallel the signaling observed with the tethered ligand, we next tested signaling induced by lipidated, glycine extended CCK4 (l-CCK4-Gly-COOH). As with tethered glycine extended CCK4, l-CCK4-Gly-COOH activated the CCK2R (Figure 5C) and lacked activity at the CCK1R (Figure 5D). Furthermore, lipidation of CCK4-Gly increased the potency of this ligand when compared with its soluble counterpart (s-CCK4-Gly-COOH) at the CCK2R. To further explore the mechanism underlying agonist mediated signaling, we evaluated the potential of a well-established CCK2R non-peptide antagonist, YM022 [26], [27], to block receptor mediated signaling. As illustrated in Figure 6, YM022 inhibits CCK2 receptor signaling induced by tethered CCK4-Gly-COOH (Figure 6A) as well as soluble and lipidated CCK4-Gly-COOH (Figure 6B). IC_50_ values are as follows: tCCK4-Gly (IC_50_ = 0.54 nM), l-CCK4-Gly-COOH (IC_50_ = 10.2 nM), and s-CCK4-Gly-COOH (IC_50_ = 0.84 nM).

To explore the combined effect of amidation and lipidation, potencies of corresponding Substance P (s-SubP-NH_2_, l-SubP-NH_2_) and CCK4 (s-CCK4-NH_2_, l-CCK4-NH_2_) analogs were also compared. Notably the combination of C-terminal amidation and peptide lipidation maintained or slightly enhanced potency relative to the EC_50_ for the endogenous amidated peptides (Table 1, Figure S2).

## Discussion

Our results demonstrate that synthetic lipidated constructs can mimic the activity of corresponding recombinant MTLs. This observation suggests a powerful two-step strategy that may be broadly applied to developing anchored peptide ligands for a wide range of targets. As a first step, an MTL with activity is identified. The recombinant nature of an MTL provides a highly efficient platform for generating and pharmacologically screening corresponding variants, thus optimizing the peptide. As a second step, the peptide is covalently attached to a lipid linker (palmitic acid-PEG) backbone enabling direct administration.

To test this two-step strategy, precursor forms of two well established peptide hormones, SubP and CCK4, were used. We noted that non-amidated SubP and glycine extended CCK4, respectively, showed agonist activity when assessed as MTLs (Figures 3 and 5). In light of the known importance of C-terminal amidation for the function of numerous biologically active peptides [14], [28], the activity of the non-amidated precursor peptides was not anticipated [13], [16]. This finding suggests that tethered precursor peptides may be active and that the requirement for post-translational modification does not necessarily preclude activity as an MTL. Thus, MTLs may provide a tool to facilitate the rapid identification of other active precursor peptides that can then be used as templates for further ligand optimization and/or the generation of mice expressing recombinant transgenic activators.

The low potency of many precursor peptides, including CCK and SubP, is due in part to the absence of C-terminal amidation as an affinity determinant [16]. We speculate that MTLs, by virtue of holding the corresponding ligand in proximity to its cognate GPCR (thus increasing the effective concentration), bypass the need for selected affinity determinants, in this case the C-terminal amide. For peptides where MTLs are active, anchoring appears to facilitate direct ligand-receptor interaction. The observed increase in potency of both SubP and CCK4 precursors with lipidation is consistent with this hypothesis. Additional modifications can be anticipated to further enhance the potency of these synthetic constructs.

Anchored precursor proteins of SubP and CCK (either as MTLs or SMALs) show receptor subtype selectivity. Like tSubP, l-SubP-COOH activates NK1 and NK3R with no activity observed at the NK2R. This phenomenon is recapitulated with CCK4: tCCK4-Gly and l-CCK4-Gly-COOH both activate the CCK2R with no activity on CCK1R. In addition to illustrating receptor subtype selectivity, these data also highlight the fact that MTLs are good predictors of the activity of SMALs. This attribute of MTLs fits well with our assertion that MTLs provide an efficient system for identifying and optimizing peptides of interest and underscores the utility of MTL-SMAL technology.

The predictive nature of MTLs both with regard to activity and subtype selectivity make them powerful tools to detect low potency activators of GPCRs that may otherwise be missed using conventional screening techniques. As an example, whereas both tSubP and l-SubP-COOH activate the NK3 receptor, no signaling is observed with the corresponding soluble ligand (s-SubP-COOH). Generalizing from this illustration, if MTL technology were used to screen for low potency ligands we can anticipate the identification of additional agonists (i.e. ones that could not be identified when screening corresponding soluble unanchored ligands). Therefore MTLs may provide a new tool to identify novel ligands for GPCRs of interest.

To better understand the mechanism underlying MTL and SMAL activity, we completed a series of experiments using well established small molecule antagonists. The ability of these compounds to inhibit the function of both genetically engineered and synthetic peptide ligands was assessed. Like their soluble counterparts, our data suggest that MTLs and SMALs act as orthosteric activators. With both SubP and CCK4, all forms of ligand activity are inhibited by CP 99994 or YM022, respectively. The IC_50_ values for antagonism at both NK1R and CCK2R are in the nanomolar range, similar to those previously reported for inhibition of amidated forms of SubP and CCK proteins [25], [27]. The ability to block SMAL activity with these highly selective antagonists further underscores the potential of these peptides as receptor specific probes.

Prior studies have examined the effects of N-terminal lipidation of the amidated cholecystokinin tetrapeptide, CCK4-NH_2_,with a focus on enhancing membrane permeability. Both acetylation and/or caproylation of CCK4-NH_2_ resulted in increased peptide stability, permeability and intestinal absorption [29]–[32]. In addition to CCK, lipidation has been utilized to modify a wide variety of peptide ligands [33]. Such modifications have led to enhanced peptide stability [34]–[36], prolonged half-life by facilitating binding to circulating albumin [34], [37], [38], and/or targeted excretion by the liver rather than by the kidney [34], [39], [40]. Additional studies have shown that lipidation can improve intestinal absorption by increasing the lipophilic properties of a ligand [30], [32], [41]. Based on existing literature, it appears that lipidation of peptides may increase, decrease, or have no influence on affinity [34], [42], [43]. MTL/SMAL technology complements and improves the above empiric approaches by providing a recombinant format in which to assess (and modify as needed) the pharmacological effects of anchoring. This may be done prior to SMAL synthesis to optimize ligand activity.

The combined MTL/SMAL approach provides a highly streamlined system for the identification of putative receptor ligands. As more targets are explored, the extent to which MTL/SMAL technology is a generalizable strategy will be better defined. Without the use of the MTL approach, many low potency peptides could be missed during screening. MTLs also offer an index of how anchoring will effect peptide activity and provide a rational approach for defining the site of synthetic anchoring, e.g. lipidation at the N or C terminus. Active MTL peptides, once identified, can be converted to SMALs using standard synthetic chemical methods. These lipid-peptide constructs show enhanced potency versus the free ligand and have the added advantage that they can be directly administered using conventional delivery methods. It can be anticipated that with libraries of cDNA encoded tethered peptides, it should be possible to identify as yet undiscovered peptides that can modulate receptors of interest. Given the power of MTL technology in peptide design as well as the potential for SMALs to enhance ligand potency and enable delivery, this combination of strategies is well-suited to expedite the development of peptide therapeutics.

## Supporting Information

Figure S1 **Chemical structure, purity, and molecular weight of synthesized amidated peptides.**
(DOCX)

Figure S2 **Lipidated amidated Substance P and lipidated amidated CCK4 activate cognate receptors NK1R and CCK2R respectively, with potencies comparable to or higher than the corresponding endogenous ligand.**
(DOCX)
