# Supplementary material for: A Two-Step Strategy to Enhance Activity of Low Potency Peptides
Source: PLoS One. 2014 Nov 12;9(11):e110502. doi: 10.1371/journal.pone.0110502 (PMC4229100; doi:10.1371/journal.pone.0110502)
Supplement: Figure S1 — Chemical structure, purity, and molecular weight of synthesized amidated peptides. (DOCX) [file pone.0110502.s001.docx]

**
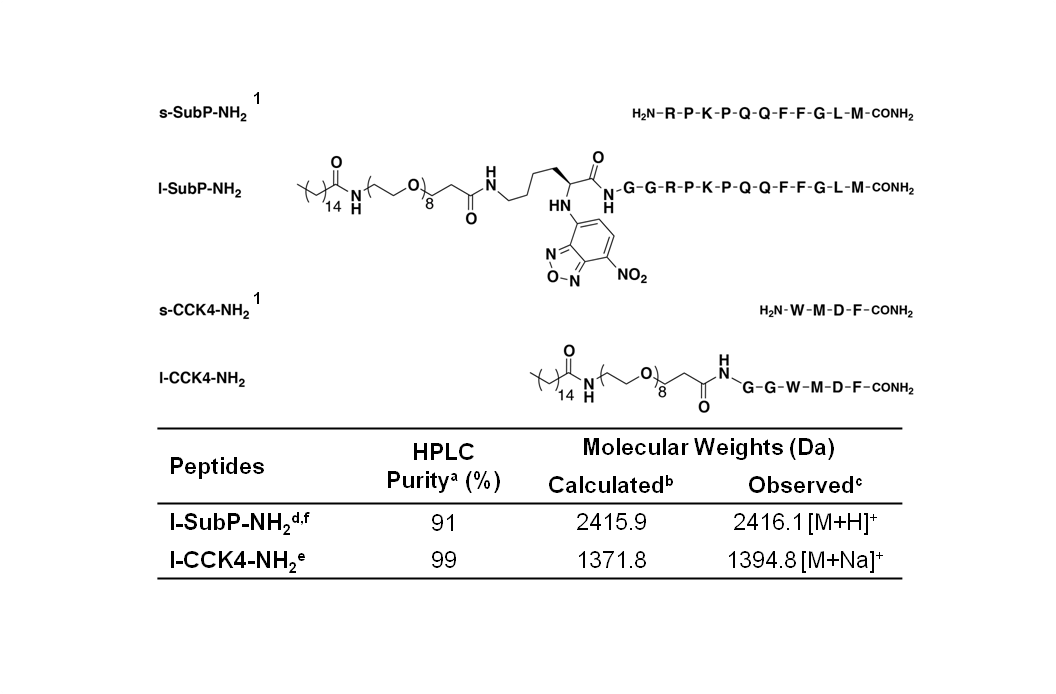
**

Figure S1. Chemical structure, purity, and molecular weight of synthesized amidated peptides.

a) Purity as determined by analytical RP-HPLC [Vydac C18, 5 μm, 4 mm × 250 mm] using a binary solvent system [A: H_2_O/CH_3_CN/TFA (99/1/0.1); B: CH_3_CN/H_2_O/TFA (90/10/0.07)] with a linear gradient of 65-80% solvent B over 20 min. The flow rate was set at 1 mL/min and elution was monitored by absorbance at 230 nm.

b) Expected molecular weights were calculated using Peptide mass calculator v3.2 and confirmed by the analysis tool in ChemBioDraw Ultra v12.0.3.

c) Observed molecular weights as determined using MALDI-TOF MS in reflectron positive mode using α-cyano-4-hydroxycinnamic acid as the matrix.

d) acetylated lysine GG spacer (Ac-Lys-GG)

e) GG spacer coupled to the *N*-terminus of the peptide before pegylation.

f) *N*^α^-amine of lysine in KGG spacer conjugated to NBD

^1^s-SubP-NH_2_ and s-CCK4-NH_2_ peptides: purchased commercially from American Peptide Company
