# Supplementary material for: A Two-Step Strategy to Enhance Activity of Low Potency Peptides
Source: PLoS One. 2014 Nov 12;9(11):e110502. doi: 10.1371/journal.pone.0110502 (PMC4229100; doi:10.1371/journal.pone.0110502)
Supplement: Figure S2 — Lipidated amidated Substance P and lipidated amidated CCK4 activate cognate receptors NK1R and CCK2R respectively, with potencies comparable to or higher than the corresponding endogenous ligand. (DOCX) [file pone.0110502.s002.docx]

Figure S2. Lipidated amidated Substance P and lipidated amidated CCK4 activate cognate receptors NK1R and CCK2R respectively, with potencies comparable to or higher than the corresponding endogenous ligand (for EC_50_ values see Table 1). l-SubP-NH_2_ (NDB labeled, see Figure S1 and methods) is fully active and more potent than s-SubP-NH_2_ on NK1R (panel A). l-CCK4-NH_2_ has similar potency to s-CCK4-NH_2_ but does not fully activate CCK2R (panel B). HEK293 cells were transiently cotransfected for 24 hours with cDNAs encoding: the designated receptor, a 5X-SRE-Luc-pest reporter gene (pGL4.33), and a β-galactosidase gene to control for transfection variability. For assessment of SMAL induced signaling, cells were stimulated with ligand for 4 hours. Luciferase activity was quantified and normalized relative to a 4 hour stimulation with 1μM soluble amidated substance P (s-SubP-NH_2_) for NK1R or 10 μM soluble amidated CCK4 (s-CCK4-NH_2_) for CCK2R. Data represent the mean ± SEM from 3 independent experiments, each performed in triplicate. Abbreviations: l-SubP-NH_2,_ lipidated amidated Substance P; s-SubP-NH_2,_ soluble amidated Substance P; NK1R, neurokinin 1 receptor; l-CCK4-NH_2,_ lipidated amidated cholecystokinin 4; s-CCK4-NH_2,_ soluble amidated cholecystokinin 4; CCK2R, cholecystokinin 2 receptor.
